# Detection bias in microarray and sequencing transcriptomic analysis identified by housekeeping genes

**DOI:** 10.1016/j.dib.2015.11.045

**Published:** 2015-11-27

**Authors:** Yijuan Zhang, Oluwafemi S. Akintola, Ken J.A. Liu, Bingyun Sun

**Affiliations:** a Department of Chemistry, Simon Fraser University, Burnaby, British Columbia, Canada; b Department of Molecular Biology and Biochemistry, Simon Fraser University, Burnaby, British Columbia, Canada

**Keywords:** Transcriptome, Microarray, Sequencing, RNA-seq, Next-generation sequencing, Housekeeping genes

## Abstract

This work includes the original data used to discover the gene ontology bias in transcriptomic analysis conducted by microarray and high throughput sequencing (Zhang et al., 2015) [1]. In the analysis, housekeeping genes were used to examine the differential detection ability by microarray and sequencing because these genes are probably the most reliably detected. The genes included here were compiled from 15 human housekeeping gene studies. The provided tables here comprise of detailed chromosomal location, detection breadth, normalized expression level, exon count, total exon length, and total intron length of each concerned gene and their related transcripts. We hope this information can help researchers better understand the differences in gene ontology-bias we discussed (Zhang et al., 2015) [1] and can encourage further improvement on these two technology platforms.

**Specifications Table**

**Table:** 

Subject area	*Biology*
More specific subject area	*Transcriptomics*
Type of data	*Excel table*
How data was acquired	*Microarray and sequencing*
Data format	*Downloaded from public domain, compiled and analyzed*
Experimental factors	*Gene identifier was unified*
Experimental features	*Analysis of gene chromosomal location, gene structure, and gene expression*
Data source location	
Data accessibility	Data is with the article

**Value of the data**

• Housekeeping genes are the most reliably detected genes in high throughput fashion that have the least detection errors for examining differences in analysis.
• The detailed value of all concerned factors including the chromosomal location, the exon count, total exon length, total intron length, normalized expression value, detection breadth are provided here in a per gene or per transcript basis such that the data can be further queried or analyzed.
• The information included here should also help further improvement on these two popular technology platforms.

## 1 Data

Table S1, chromosomal location of housekeeping (HK) genes exclusively detected by MA alone, sequencing alone, as well as jointly. Table S2, exon count, total exon length, total intron length, and GC content of HK genes exclusively detected by MA alone, sequencing alone, as well as jointly. Table S3, detection breadth and the normalized maximum expression quantity of each HK gene exclusively detected by MA alone, sequencing alone, as well as jointly.

## 2 Experimental design, materials and methods

The data included here were downloaded from 15 published human housekeeping studies, i.e. Warrington [2], Hsiao [3], Eisenberg_03 [4], Tu [5], Dezso [6], She [7], Chang [8], Shyamsundar [9], Zhu_MA, Zhu_EST [10], Podder [11], Reverter [12], Ramskold [13], Eisenberg_13 [14] and Fagerberg [15], in which nine studies used microarray (MA) analysis, i.e. Warrington [2], Hsiao [3], Eisenberg_03 [4], Tu [5], Dezso [6], She [7], Chang [8], Shyamsundar [9], Zhu_MA, and the rest used sequencing analysis. The gene identifiers used in different studies were first converted to entrez gene ID using Database for Annotation, Visualization and Integrated Discovery (DAVID) v6.7 (http://david.abcc.ncifcrf.gov/) [16], [17] as detailed in [1], [18]. The chromosomal location was queried against National Center for Biotechnology Information (NCBI) (http://www.ncbi.nlm.nih.gov). Genes with unknown genome locations were removed. The obtained entrez gene list was further converted to Refseq mRNA IDs using DAVID, and the Refgene information on exon count, exon starting and ending position as well as the coding sequences were obtained by querying the Refgene information from University of California, Santa Cruz (UCSC) genome browser (http://genome.ucsc.edu/index.html) against the latest human genome assembly (GRCh38) [19]. The total intron length was calculated by the total gene length minus total exon length. The GC content was deduced by the coding sequence only. Again transcripts could not be mapped to Refgene in UCSC database, and those without exon count or exon starting or ending information as well as sequencing information, were removed from the table. The expression quantity was collected from Chang [8], Eisenberg_03 [4], She [7], Warrington [2], Shyamsundar [9] and Fagerberg [15]. The raw expression quantity was first normalized against the maximum value in each individual list to make them comparable. For entrez genes having multiple quantification values in a single list (for example in cases where a single entrez gene ID was mapped to several IDs, each IDs in that particular study had an expression value), the maximum normalized expression value was used. The detective breadth (DB) [1], [18] described the number of studies, in which a HK gene had been identified. For example, if a gene was detected in 8 out of 9 MA studies, its DB value would be 8, and similarly if a gene was detected in 5 out of 6 sequencing studies, its DB value would be 5.
